# The Efficacy of Landscape-Level Conservation in Changbai Mountain Biosphere Reserve, China

**DOI:** 10.1371/journal.pone.0095081

**Published:** 2014-04-17

**Authors:** Jianliang Zhang, Fangzheng Liu, Guofa Cui

**Affiliations:** College of Nature Conservation, Beijing Forestry University, Beijing, China; National University of Singapore, Singapore

## Abstract

Anthropogenic landscape alteration is rather common in many protected areas (PAs), jeopardizing the efficacy of PAs conservation. However, the general consensus is that PAs still remain effective in habitat conservation. To assess the efficacy of landscape-level conservation, we examined landscape alterations in the Changbai Mountain Biosphere Reserve (CMBR), which was established in 1960 as a “flagship” protected area in China. Based on analyses of high-resolution satellite images and data of forest inventory, field survey and interview, we developed two new indexes to assess the efficacy of landscape conservation, i.e. the quality index of protected landscape and the interference index of anthropogenic landscape. From 1993 to 2012, the quality index increased from 74.48 to 75.50, and the interference index decreased from 0.49 to 0.06, suggesting that the overall quality of protected landscape improved and the degree of anthropogenic interference decreased in CMBR. The increase in landscape quality was mainly due to the progressive vegetation recovery of previous cutover land in the windthrow area, the cease of the use of the cultivated land, and the amelioration of spatial pattern of protected landscape. We conclude that the current landscape conservation methods used in CMBR are effective, and the method we developed has the potential to be used to assess the efficacy of landscape-level conservation in nature reserves worldwide.

## Introduction

Habitat reduction and fragmentation are the leading causes of biodiversity loss worldwide [1], [2], and establishing protected areas (PAs) or natural reserves is the principal defense [3]–[5]. Prior to 2012, there had been 130,709 PAs worldwide, covering more than 12.9% of total land surface [6]. In PAs, two types of landscape are identified, i.e. protected landscape and anthropogenically interfered landscape (thereafter referred to as “interfered landscape”) [7], [8]. The former refers mainly to natural landscape and also includes some artificial landscape that is beneficial to wildlife [8]. The latter is frequently interfered by human activities that often disturb and damage natural ecosystems and wildlife habitats [9]. Currently interfered landscape is rather common in PAs [10]–[12], and expected to increase because human population directly adjacent to PAs continues to expand [13], [14]. However, the general consensus is that PAs still remain effective in habitat conservation [5], [15]. Therefore, it is highly necessary to examine changes in the quality of protected landscape and the degree of anthropogenic interference to assess the efficacy of landscape-level conservation in nature reserves [16]–[18].

In China, “Rescue” efforts had resulted in 2640 nature reserves (excluding those in Hong Kong, Macao, and Taiwan) before 2012 [19]. Nevertheless, the absence of systematic conservation planning [20] and the continued adoption of unsustainable policies [21], [22] have resulted in uncontrolled utilization of resources in nature reserves. For example, rapid development of ecotourism industries has led to dramatic changes in landscape type [23]–[25]. Quantitative information about efficacy of landscape conservation for China's nature reserves is scant, and there are no studies examining landscape changes in PAs based on high-resolution satellite images.

The combination of remote sensing and geographic information system has created a powerful analytical method for exploring landscape dynamics and testing the efficacy of PAs [17], [18], [26]–[28]. Nevertheless, due to the limitation of spatial resolution in satellite images (≥30 m, generally), classifications in PAs were simple and usually consisted of only forest and non-forest categories [10], [12], [29], [30], and only large area of deforestation could be detected. Landscape changes driven by human activities at smaller scales (<30 m) in PAs such as residential houses, small hydropowers, ditches, industrial and commercial facilities were often incognizable. In recent years, the occurrence of satellite images with higher spatial resolution has allowed for a more accurate recognization of interfered landscape at smaller scales. However, there is still lack of a comprehensive evaluation method combining high-resolution image data and other sources of data (forest inventory, field survey and interview) to quantify the overall quality of protected landscape and the degree of anthropogenic interference.

To examine the efficacy of landscape conservation, we develop two new indexes: the quality index of protected landscape and the interference index of anthropogenic landscape. Based on data of high-resolution satellite images, forest inventory, field survey, and interview, landscape was first classified into different types and each type was assigned to a value according to its contribution to habitat protection. Then the two indexes were constructed to quantify the overall quality of protected landscape and the degree of anthropogenic interference. We adopted this method to assess the efficacy of landscape conservation within the Changbai Mountain Biosphere Reserve (CMBR). CMBR was selected as it is one of the earliest nature reserves established in China and classified as a demonstration [31]. The evaluation method applied in CMBR has the potential to be used in other nature reserves in China as well as those in other countries. The objectives were to (1) provide a quantitative method for assessing efficacy of landscape-level conservation, (2) assess the efficacy of landscape conservation in CMBR using this method, and (3) recommend protection and management strategies.

## Materials and Methods

### Ethics statement

This study obtained relevant permissions from the administrative bureau of CMBR, and was conducted under the Nature Reserve Regulation and the Wildlife Protection Law of the People's Republic of China.

### General situation of the research area

**Figure 1:**
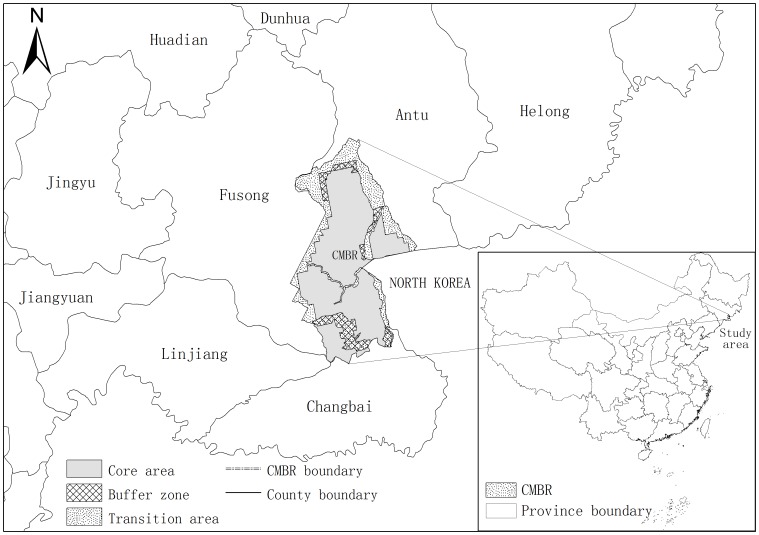
Location of Changbai Mountain Biosphere Reserve (CMBR). The CMBR is located in the northeast of China. It borders the Democratic People's Republic of Korea (DPRK) to the southeast.

CMBR is located in the central and eastern part of Jilin Province, China (127°42′55″–128°16′48″E, 41°41′49″–42°25′18″N; Fig. 1). It borders the Democratic People's Republic of Korea to the southeast. According to the PAs model launched by UNESCO's Man and Biosphere Program [32], the reserve is divided into three functional zones, i.e. a core area, a buffer zone, and a transition area, accounting for 65.3, 10.2 and 24.5% area of CMBR, respectively (Fig. 1). The core area where harvesting and poaching are absolutely prohibited is composed of old-growth forests. The buffer zone, surrounding the core area, is established to prevent the core area from human disturbances. The transition area is outside the buffer zone, and used as an area for re-establishment of endemic species, ecotourism and bases for in situ reproduction of natural resources.

Under the influence of monsoon climate, the Changbai Mountain area has a temperate continental climate with dry and windy springs, warm and rainy summers, cool and foggy autumns, and cold and long winters. The mountain exhibits high variation in climate conditions due to an expansive elevation gradient, ranging from 700 to 2691 m. The base of the mountain has the typical warm temperate climate, whereas higher elevations have complex, changeable near polar climates [33]. Thus, an obvious vertical zonation of vegetation is present, forming five vegetation zones: broad-leaved forest (<720 m), coniferous and broad-leaved mixed forest (720–1,100 m), coniferous forest (1,100–1,700 m), *Betula ermanii* forest (1,700–2,000 m) and alpine tundra (>2,000 m) [34]. CMBR is rich in biodiversity due to its diverse topography, climate, and ecosystems. For example, there are 9 species of amphibians, 12 reptiles, 24 fish, 56 mammals, 230 birds, and 1255 insects [35]. CMBR is also home to 430 fungi, 200 lichens, 311 mosses and liverworts, 78 ferns, 11 gymnosperms, and at least 1325 angiosperms [36].

### Sources of data

#### Direct access from CMBR management organization

We obtained the reserve boundary maps, functional zone maps, road maps, data of forest inventory in 1993, and records of nature reserve administration. In the data of forest inventory, different landscape types were delineated using aerial photographs (1∶34,000), LandSat TM image and topographic data (1∶25,000), in combination with fixed sample plots. The different landscape types included natural forest, artificial forest, sparse forest, shrub forest, young afforested land, grassland, cutover land, forestry facilities (e.g. construction land and road), and other land types (e.g. cultivated land and bare land). Inspection and acceptance protocols for the data were in accordance with the “Quality Management Method of the Survey of the Forest Resources of Jilin Province” [37]. The system of inspection and acceptance was divided into three levels: survey team, office and bureau check. The proportions of field check by survey team, office and bureau were 10, 8, and 5%, respectively. Before finishing the field survey, sampling check was used to examine the field survey quality by office and bureau. All survey quality of every survey team was qualified to reach to accuracy requirement. The proportions of indoor check by survey team and office were 100 and 65%, respectively. The overall accuracy of the data was 96.2% according to bureau check [37].

#### Remote sensing imagery

We obtained the ZY-1 02C HR orthorectified panchromatic image (spatial resolution 2.36 m), ZY-1 02C P/MS multi-spectral image (spatial resolution 10 m) and LandSat TM image in 2010, with the spatial resolution of 30 m. Both ZY-1 02C HR and P/MS images were acquired in 2012, and provided by China Center for Resource Satellite Data and Application. The TM image was obtained from International Scientific Data Service Platform. The P/MS and TM images were georeferenced to HR orthorectified panchromatic image with submeter accuracy. The ZY-1 02C fusion image (spatial resolution 2.36 m) was generated from HR and P/MS images mentioned above, by using Gram-Schmidt Spectral Sharpening Method.

#### Field survey data

**Figure 2:**
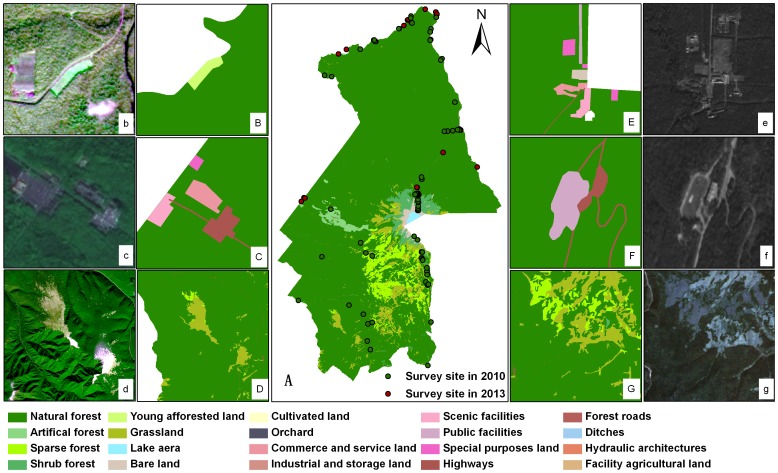
Composite map of landscape classification for CMBR in 2012. (A) Full map of landscape classification, (B) Young afforested land on northern slope, (b) The ZY-1 02C fusion image of B, (C) Scenic facilities, commerce and service land, highways, and special purposes land near the western gate, (c) ZY-1 02C fusion image of C, (D) Natural forest and grassland on southern slope, (d) ZY-1 02C fusion image of D, (E) Scenic facilities, commerce and service land, special purposes land near the northern gate, (e) The HR panchromatic image of E, (F) Highways and public facilities in the U-shaped valley, (f) The HR panchromatic image of F, (G) Natural forest, sparse forest and grassland on the southern slope, (g) The TM image of G.

Field data of different types of landscape in CMBR were collected in August 2010, using a portable GPS to record geographic locations (including longitude, latitude and elevation) in 71 surveyed sites (Fig. 2A). These data were used to establish interpretation symbol for image classification. To classify the interfered landscape such as residential houses, small hydropowers, ditches, industrial and commercial facilities, the special purposes of such landscape were surveyed in the field in May 2013 and used as supplementary information. A total of 23 sites was survived (Fig. 2A). Actual width of the interfered landscape with linear feature (such as road and ditches) was measured in the field, when the width of the landscape was less than 10 m. In addition, vegetation recovery in the windthrow area above altitude of 1,500 m was investigated in 10 plots (10 m×10 m), in which canopy closure, plant species composition, and seedlings regeneration were recorded.

#### Interviews with regional stakeholders (e.g. CMBR staff and local residents)

The interviews focused on changes in the interfered landscape, especially changes in type, area, and spatial distribution from 1993 to 2012.

### Landscape classification

With reference to Current Land Use Classification [38], the landscape in CMBR was initially divided into natural, and artificial landscape. The former included six types of landscape: natural forest, sparse forest, shrub forest, grassland, lake area, and bare land. The latter included artificial forest, young afforested land, cutover land, cultivated land, orchard, commerce and service land, industry and storage land, residential land, scenic facilities, public facilities, special purposes land (e.g. military), highways, forest roads, ditches, hydraulic architectures, and facility agricultural land.

*Classification of natural landscape*. Based on the data of forest inventory in 1993, the classification layer of natural landscape of CMBR in 1993 was established using ArcMap 10.0 software according to the six types of landscape mentioned above. In contrast to the ZY-1 02C image, the LandSat TM image of CMBR was of high quality (no influence of clouds and snow), and could provide more band combination to identify different types of vegetation. The 30 m spatial resolution satisfied accuracy requirements for the classification of a nature reserve covering a total area of nearly 200,000 ha. Therefore, the TM image was adopted for the classification of natural landscape in 2012. Using the ENVI 5.0 software, an Example-Based Object-oriented Classification method was employed to classify the natural landscape (See Text S1 for the detailed processes of classification). The confusion matrix was computed using the validation samples selected from the ZY-1 02C fusion image and HR panchromatic image, and the classification accuracy was tested. The overall accuracy was 92.3%, and the Kappa Coefficient was 0.9. The classification accuracy for each type of natural landscape was shown in Table S1.

#### Extraction of artificial landscape

The ZY-1 02C fusion image and HR panchromatic image supplemented with the field survey data were used to classify the artificial landscape. Visual interpretation (i.e. direct delineation and interpretation of the images) was applied. An artificial landscape presented in 2012 was extracted by the ArcMap10.0 software to establish the layer of artificial landscape for that year. The central line was drawn on images of the linear landscape with width less than 10 m. Their patches were generated using bilateral buffering, according to the measured width.

Data from the artificial forest, young afforested land, cutover land, land for the forestry facilities, and other lands extracted from the data of forest inventory in 1993 were used, and supplemented with the artificial landscape data interpreted in 2012. In addition, the variation of artificial landscape for 1993-2012 was obtained via interview. The distribution of artificial landscape in 1993 was reconstructed, and the layer of artificial landscapes of 1993 was generated.

#### Establishment of composite landscape map

The classification layers of the natural, and artificial landscape were merged by ArcMap10.0. A topological approach was used to remove gaps, and to process overlapping surfaces. Composite landscape layers for 1993 and 2012 were then established. The landscape classification composite map for CMBR in 2012 is presented in Fig. 2.

### The ecological level of landscape

Based on the ecological function and role in habitat conservation, the natural and artificial landscape was further divided into three functional types: protected landscape, interfered landscape, and neutral landscape. The golden section method was used to classify the ecological level of landscape into seven grades: 1.00, 0.62, 0.38, 0, −0.38, −0.62 and −1.00 (Table 1). The golden section method was a typical algorithm in selecting optimization based on Golden Section Theory [39], and it used the critical value of 0.62 and 0.38 to divide the given interval. A positive value was assigned to the protected landscape; the greater the value, the higher the ecological level. A negative value was assigned to the interfered landscape; the greater the absolute value, the smaller the ecological level (i.e. the higher the interference degree). The value 0 was assigned to the neutral landscape.

**Table 1:** Value of ecological level of different landscape in CMBR.

Landscape type	Attribute	Ecology level
Natural forest, Lake area	Protected landscape	1.00
Sparse forest, Shrub forest	Protected landscape	0.62
Grassland, Artificial forest, Young afforested land	Protected landscape	0.38
Bare land	Neutral landscape	0
Cutover land, Cultivated land, Orchard	Interfered landscape	−0.38
Residential land, Scenic facilities, Public facilities, Forest roads, Facility agricultural land	Interfered landscape	−0.62
Commerce and service land, Industry and storage land, Special purposes land, Highways, Ditches, Hydraulic architectures	Interfered landscape	−1.00

### Quality index of protected landscape

We developed the quality index of protected landscape (*Q*) as follows:
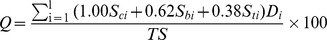
where *S_ci_*, *S_bi_* and *S_ti_* are the area of protected landscape of type *i* in the core area, in the buffer zone and in the transition area, respectively, 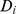 the value assigned to the ecological level of protected landscape of type *i*, *TS* the total area of the nature reserve, and *l* the number of protected landscape types. The constants 1.00, 0.62 and 0.38 represent the weights of the core area, buffer zone and transition area, respectively.

The value of *Q* ranges from 0 to100. If there is no protected landscape in the nature reserve, then *Q* equals 0; if landscape in the nature reserve is all protected landscape, all distributed in the core area and with the highest ecological level (1.00), then Q equals 100. By comparing the *Q* values in 1993 with those in 2012, changes in the quality of protected landscape in the nature reserve could be quantified.

### Interference index of anthropogenic landscape

We also developed the interference index of anthropogenic landscape (*I*) as follows:
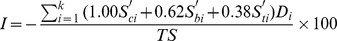
where *S*′*_ci_*, *S*′*_bi_* and *S*′*_ti_* are the area of interfered landscape of type *i* in the core area, in the buffer zone and in the transition area, respectively, 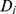 the value assigned to the ecological level of interfered landscape of type *i*, and *k* the number of interfered landscape types.

The value of *I* ranges from 0 to 100. If there is no interfered landscape in the nature reserve, then *I* = 0. If landscape in the nature reserve is all interfered landscape, all distributed in the core area and with the lowest ecological level (−1.00), *I* = 100. The greater the value is, the higher is the interference degree. By comparing the *I* values in 1993 and in 2012, the change in the degree of anthropogenic interference in the nature reserve could be quantified.

### Alterations of landscape area of the same types

We calculated the alteration rate of landscape area in the natural reserve as:
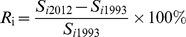
where *R_i_* is the alteration rate of the area of landscape type *i*, *S_i2012_* the area of landscape type *i* in 2012, and *S_i1993_* the area of landscape type *i* in 1993.

### Alterations of landscape area of different types

Based on the composite landscape maps in 1993 and 2012, we calculated the area transfer matrix between different landscape types using the ENVI 5.0 software. The proportion of area transformed to (*A_p→q_*) and from (*B_p←q_*) other types of landscape proportion were calculated as:
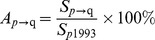


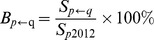
where *A_p→q_* is the proportion of area transformed to other type of landscape (i.e. the proportion of the area of landscape type *p* in 1993 transformed into landscape type *q* in 2012), *S_p→q_* the area of landscape type *p* in 1993 transformed into landscape type *q* in 2012, *S_p1993_* the area of landscape type *p* in 1993, *B_p←q_* the proportion of the area newly added (i.e. the proportion of the area of landscape type *p* in 2012 transformed from landscape type *q* in 1993), *S_p←q_* the area of landscape type *q* in 1993 transformed into landscape type *p* in 2012, and *S_p2012_* the area of landscape type *p* in 2012.

### Alterations of spatial pattern of protected landscape mosaic

Using the ArcMap10.0 software, protected landscape with ecological levels of: (1) 0.38 and above (including seven types: natural forest, lake area, sparse forest, shrub forest, grassland, artificial forest, young afforested forest), (2) 0.62 and above (including natural forests, lake area, sparse forest and shrub forest), and (3) 1.00 (natural forest and lake area) were merged into one landscape mosaic, respectively. Integrating different habitat resources into a complete pattern made it possible to analyze the overall influence brought to the survival species due to variations in spatial pattern of habitats.

The pattern indexes, including the number of patches (*NP*), edge density (*ED*), perimeter-area fractal dimension (*FR*), division index (*DI*), and connectivity index (*CON*), were computed by the FragStats 4.0 software, the equations of the indexes were detailed in (Text S2). *NP* is fundamentally important to a number of ecological processes. If the target landscape mosaic area increases, the rise of *NP* may reflect the early stage in progressive succession of vegetation. As vegetation recovery proceeds further, *NP* may hold constant or decrease because of the merger of the neighbor landscape. *ED* and *FR* quantify the edge size and the degree of shape complexity of the target landscape mosaic, and are suggestive of anthropogenic interference affecting the target landscape mosaic across their edges at a wide range of scales [40]. *DI* refers to the tendency of the target landscape mosaic to be spatially aggregated and reflects the degree of habitat fragmentation [41]. *CON* refers to the degree to which a landscape facilitates or impedes ecological flows (e.g., the movement of organisms among habitat patches) [42]–.

## Results

### Changes in the quality of protected landscape

The quality index of protected landscape increased from 74.48 in 1993 to 75.50 in 2012, indicating that the overall quality of protected landscape in CMBR improved. From 1993 to 2012, the total area of protected landscapes increased from 191,862.5 ha (97.8% of the total area of the reserve) to 194,567.3 ha (99.2%; Table 2). The natural forest area increased from 168,693.0 ha to 170,898.4 ha, and the increased area was mainly transformed from sparse forest (1,555.1 ha) and cutover land (1,300.9 ha; Table S4). The area of sparse forest increased from 7,505.2 ha to 7,968.0 ha by 6.2%. The 1,140.4 ha of cutover forest and 1,181.1 ha of grassland were transformed into sparse forest (Table S4). The total area of protected landscape in the core area, buffer zone, and transition area all increased to some extent (Table 2). The total area of protected landscape in the core area increased from 127,893.9 ha to 129,743.7 ha, and increased by 846.5 ha in the transition area. The area of sparse forest in the buffer zone, and the transition area increased by 68.6% and 64.9%, respectively; the area of the natural grassland in the transition area increased by 10.1% (Table S3).

**Table 2:** Total area and percentage of protected landscape and interfered landscape in each functional zone.

	Protected landscape					Interfered landscape				
	Area in 1993/ha	Area in 2012/ha	Percentage in 1993/%	Percentage in 2012/%	Variation ratio/%	Area in 1993/ha	Area in 2012/ha	Percentage in 1993/%	Percentage in 2012/%	Variation ratio/%
Biosphere reserve	191862.5	194567.3	97.8	99.2	1.4	3033.5	320.8	1.5	0.2	−89.4
Core area	127893.9	129743.7	98.0	99.4	1.4	1894.9	57.8	1.5	0.0	−96.9
Buffer zone	18036.3	18045.3	99.2	99.2	0.0	53.1	39.7	0.3	0.2	−25.2
transition area	45929.4	46775.9	96.7	98.5	1.8	1084.8	222.2	2.3	0.5	−79.5

### Changes in the interference degree of interfered landscape

The interference index of anthropogenic landscape decreased from 0.49 in1993 to 0.06 in 2012, suggesting that the degree of anthropogenic interference decreased. From 1993 to 2012, the total area of interfered landscape in the nature reserve was reduced from 3,033.5 ha to 320.8 ha, with a variation rate of −89.4% (Table 2). Cutover land, cultivated land, industry and storage land, residential land, forest roads and hydraulic architectures decreased greatly. The area of cutover land transformed to natural forest was 1,300.9 ha, accounting for 49.0%, and about 43.0% of cutover land was transformed into the sparse land (Table S4). In addition to those transformations, there were still 206.6 ha of cutover land transformed to grassland (Table S4). The area of cultivated land transformed to artificial forest was 48.1 ha (56.6%), and there were 23.7 ha (27.9%) cultivated land transformed to grassland, shrub forest, sparse forest and other types of protected landscape (Table S4).

The total area of interfered landscape in the core area, buffer zone and transition area significantly decreased. The area of interfered landscape in the core area was 1,894.9 ha in 1993, and reduced to only 57.8 ha in 2012 (Table 2). The area of interfered landscape in the buffer zone was the lowest both in 1993 and in 2012 (Table 2), and primarily used for transportation. The construction land (i.e. commerce and service land, industry and storage land, residential land, scenic facilities, public facilities, special purpose land, highways, forest roads, hydraulic architectures and facility agricultural land), cultivated land, and orchard in the transition area were significantly larger than those in the core area and buffer zone. The total area of the three types mentioned above was 258.2 ha in 1993, accounting for 23.8% of the total area of interfered landscape. Among these, the total area of construction land increased by 26.9%. Especially, the area of commerce and service land increased significantly from 2.6 ha to 13.4 ha (Table S3).

### Changes in spatial pattern of protected landscape mosaic

**Figure 3:**
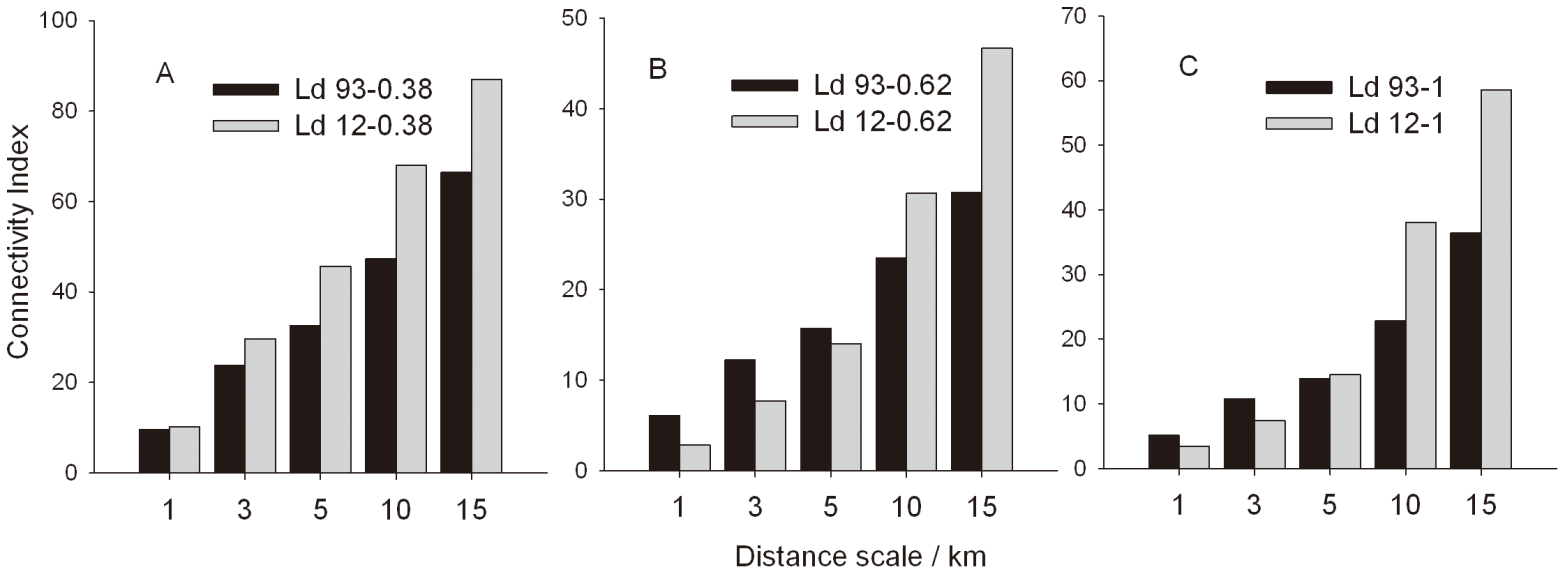
Connectivity index of protected landscape mosaic. Ld 93-0.38, Ld 93-0.62, Ld 93-1 represents the landscape mosaic composed by protected landscape with ecological levels ≥0.38, ≥0.62 and ≥1 in 1993, respectively. Similarly, Ld12-0.38, Ld12-0.62, Ld12-1 represents the landscape mosaic composed by protected landscape with ecological levels ≥0.38, ≥0.62 and ≥1 in 2012, respectively.

From 1993 to 2012, *NP* of protected landscape mosaic (with an ecological level of ≥0.38) increased from 281 to 345, *ED* decreased from 6.70 to 6.08, *FR* decreased from 1.74 to 1.53, and *DI* did not change significantly (Table S2). The spatial connectivity of protected landscape at scales of 1, 3, 5, 10 and 15 km all significantly increased (Fig. 3A). *NP* of protected landscape mosaic (with an ecological level of ≥0.62) increased from 271 to 288, whereas *ED* slightly increased from 14.57 to 14.61. The overall shape became simpler, and the degree of fragmentation did not change significantly (Table S2). The spatial connectivity decreased at smaller scales (1, 3, and 5 km), but increased at larger scales (10 and 15 km; Fig. 3B).

The natural forest and lake area both had an ecological level of 1. As the lake area experienced little or no change, variation in landscape pattern was mainly driven by the change in the area of natural forest. From 1993 to 2012, *NP* of natural forest increased from 333 to 456, and its *ED* increased from 16.24 to 16.39. The degree of fragmentation increased, and *DI* increased from 0.56 to 0.61. The spatial connectivity of natural forest decreased at smaller scales (1, and 3 km), but increased at scales of ≥5 km (Fig. 3C).

## Discussion

### Efficacy of landscape conservation

Changes in the quality index of protected landscape and the interference index of anthropogenic landscape indicate that from 1993 to 2012 the quality of protected landscape increased and the degree of anthropogenic interference decreased. These results suggest that the protection and management measures used in CMBR are generally effective.

Four reasons account for the effectiveness of landscape conservation in CMBR. First, from 1993 to 2012, the cutover land in the windthrow area gradually resembled the natural landscape following the 20 years of recovery. The progressive succession of large areas of this cutover land resulted in the formation of natural forest and sparse forest. This is also the primary reason why the degree of anthropogenic interference decreased and the area of protected landscape increased.

Second, following the recovery, canopy closure in some parts of sparse forest in the windthrow area increased gradually to over 0.2 (i.e. the threshold value for forming forest in China [46]), suggesting the establishment of forest. In addition, the pioneer tree species also colonized within 1181.1 ha of natural grassland at lower elevations (Table S4), thus forming sparse forest through progressive succession during the 20 years.

Third, the area of cultivated land in the transition area markedly decreased. The cultivated land area in 1993 was primarily distributed along the northern boundary of the biosphere reserve, and contracted to local residents to reclaim. By 2012, the administrative authority of CMBR no longer allowed such use, and most of such cultivated land was abandoned and transformed to artificial forest, grassland, shrub forest, sparse forest and other types of protected landscape.

Forth, the spatial pattern of the protected landscape mosaic (with an ecological level of ≥0.38), which was important habitat for animals and plants, was optimized during the 20 years. The edge effect of protected landscape mosaic was weakened and its shape became simpler, as indicated by the decreased *ED* and *FR*. This further implies that the possibility of anthropogenic interference attempts across borders decreased. The increased spatial connectivity may suggest that it became easier for animals to migrate, feed or survive in the landscape mosaic in 2012 compared to that in 1993 [44]–[45]. For natural forest, the edge density and degree of fragmentation increased in the course of the 20 years, indicating the occurrence of natural succession of cutover land, grassland and sparse forest, and subsequent formation of many small patches of natural forest. These suggest that an increase in edge density and fragmentation must occur at the earlier stage of vegetation recovery. But as the succession proceeds further, the edge density and degree of fragmentation will decrease [47].

### Challenges for landscape conservation

Although most of the cutover land in the windthrow area was transformed into sparse forest and natural forest through progressive succession, there were still 206.6 ha of cutover land transformed into grassland (Table S4), distributed mainly above 1500 m asl. on the south slope in the biosphere reserve. This type of grassland may not represent the original high-quality vegetation, but may well represent the end point in vegetation succession. Field investigation indicates that the plant species in this grassland were members of the *Gramineae*, *Compositae* and *Cyperaceae* families. *Deyeuxia angustifolia* and *Synurus deltoides* were dominant species in this plant community. There were few pioneer tree species colonizing this area, although there was no lack of tree provenance. Luxuriant growth of herbaceous plants can reduce the chance of tree seed to in contact with the soil, thereby reducing germination rates. Intensive coiling of grass roots (usually 5–10 cm thick) also prevents the rooting of tree seedlings. Areas with thin, barren soil and arid climate are also unfavorable for the regeneration of trees [48]. Therefore, it is difficult for progressive vegetation succession to occur in grassland that was transformed from cutover land.

The form of human interference changed fundamentally over the past 20 years, from timber collection in windthrow area to facility and transportation corridor construction for supporting a thriving tourism industry. This change was evident from the observed increase in construction land, especially the increase of commerce and service land, scenic facilities, public facilities and highways (Table S3). In 1990s, tourist activity in CMBR was concentrated on the northern slope of Changbai Mountain. In about 2000, an additional tourist route opened on the western slope, and after the Administration Committee of Changbai Mountain was established in 2005, new tourist routes on the southern slope were also opened. An airport was even constructed outside CMBR.

Promoting the tourism industry in CMBR also resulted in an increased demand for support services, and thus, construction of numerous hotels, roads, parking lots, and scenic promenade occurred [24]. Such type of construction permanently destroyed the original vegetation, and the habitats, thereby lowering the ecosystem value of the landscape. Improper disposal of white pollutants, solid waste, and sewage also resulted in ecological destruction [49]. In addition, the number of tourists increased from 0.20 million in 2000 to 1.42 million per year in 2011 [25], and the demand on resources such as firewood and vegetables, increased rapidly [30]. Such activity also greatly increased the occurrence of fire [31]. Therefore, the development of tourism has negatively affected habitat conservation effort in CMBR.

Land for hydraulic architectures in CMBR includes hydropower stations, a dam, and other water conservancy facilities. From 1993 to 2012, the total area of the land for this use was reduced (Table S3), primarily due to the demolition of several hydropower stations, however, in 2012, there were still 6.8 ha of land dedicated to hydraulic architecture use, including 6 large hydropower stations and 3 ditches, which had a total length of 4.4 km and cover an area of 3.3 ha. These dams, hydropower stations, and ditches were all constructed along natural rivers. Thus the development and utilization of water resources affects the migration and spawning of fish [50], and severely interferes with waterfowl, and amphibian habitat [51]–[53].

### Suggestions for landscape protection

Artificial stimulation (e.g. weed removal and soil surface exposure) may help tree seed germination, and subsequent natural regeneration, because the grassland (above 1500m asl.) transformed from cutover land is not as robust as original natural forest.

The continued development of tourism in this region should follow the eco-tourism model and all visitor services should be provided outside of CMBR. Since the hydraulic architecture uses seriously affect protection of biodiversity, it may be beneficial to implement a program to gradually demolish these facilities or re-locate those to less-sensitive ecosystems.

The administrative departments of the CMBR should formulate specific regulations, and strengthen the enforcement of those regulations (as with national regulations for the protection of nature reserve). All tourism, production and business activities within the core area and buffer zone should be prohibited. The exploration, planning and resource development in the transition area (especially the development of the construction projects) must: (1) be endorsed by the administrative bureau; (2) follow a strict environmental impact assessment system, and (3) not commence until approved by higher level department of forestry.

## Conclusions

We conclude that landscape conservation in CMBR is effective, although there still exists some challenges. The methods developed in this study have the potential to be used to assess the efficacy of landscape conservation in PAs worldwide. Ideally it would be much more robust to compare also landscape changes in the surrounding, non-protected area from 1993 to 2012. But, unfortunately, data of forest inventory in 1993 in surrounding area of CMBR is not available.

## Supporting Information

File S1 Table S1 Classification accuracy of each type of natural landscape in 2012. Table S2 Spatial pattern characteristics of protected landscape mosaic. Table S3 Variation of area of landscape in CMBR and in each functional zone from 1993 to 2012. Table S4 Area transfer matrix of landscape type in CMBR from 1993 to 2012. Text S1 Specific processes of classification for natural landscape. Text S2 Equations of pattern indexes.
(ZIP)
