# Supplementary material for: The Efficacy of Landscape-Level Conservation in Changbai Mountain Biosphere Reserve, China
Source: PLoS One. 2014 Apr 17;9(4):e95081. doi: 10.1371/journal.pone.0095081 (PMC3990618; doi:10.1371/journal.pone.0095081)
Supplement: File S1 — Table S1 Classification accuracy of each type of natural landscape in 2012. Table S2 Spatial pattern characteristics of protected landscape mosaic. Table S3 Variation of area of landscape in CMBR and in each functional zone from 1993 to 2012. Table S4 Area transfer matrix of landscape type in CMBR from 1993 to 2012. Text S1 Specific processes of classification for natural landscape. Text S2 Equations of pattern indexes. (ZIP) [file pone.0095081.s001.zip › Table S3.doc]

**Table S3**. Variation of area of landscape in CMBR and in each functional zone from 1993 to 2012

| **Landscape type** | **Area in biosphere reserve/ha** | | | **Area in core area/ha** | | | **Area in buffer zone/ha** | | | **Area in transition area/ha** | | |
| --- | --- | --- | --- | --- | --- | --- | --- | --- | --- | --- | --- | --- |
|  | **In 1993** | **In 2012** | **Variation ratio/%** | **In 1993** | **In 2012** | **Variation ratio/%** | **In 1993** | **In 2012** | **Variation ratio/%** | **In 1993** | **In 2012** | **Variation ratio/%** |
| 1. Natural forest | 168693.0 | 170898.4 | 1.3 | 108243.0 | 109887.7 | 1.5 | 16938.2 | 17005.5 | 0.4 | 43509.0 | 44003.0 | 1.1 |
| 2. Artificial forest | 224.6 | 2135.9 | 851.0 | 126.1 | 1682.6 | 1234.4 | 0.0 | 2.1 | - | 98.5 | 451.2 | 358.1 |
| 3. Sparse forest | 7505.2 | 7968.0 | 6.2 | 7002.0 | 7134.4 | 1.9 | 97.1 | 163.8 | 68.6 | 406.0 | 669.7 | 64.9 |
| 4. Shrub forest | 4902.4 | 4857.4 | -0.9 | 3495.4 | 3475.7 | -0.6 | 479.3 | 481.1 | 0.4 | 927.6 | 900.6 | -2.9 |
| 5. Young afforested land | 1874.0 | 5.8 | -99.7 | 1562.1 | 0.0 | -100.0 | 2.1 | 0.0 | -100.0 | 309.8 | 5.8 | -98.1 |
| 6. Cutover land | 2653.6 | 0.0 | -100.0 | 1820.8 | 0.0 | -100.0 | 6.1 | 0.0 | -100.0 | 826.7 | 0.0 | -100.0 |
| 7. Grassland | 8245.4 | 8283.9 | 0.5 | 7060.2 | 7158.2 | 0.0 | 518.6 | 391.9 | -24.4 | 666.5 | 733.7 | 10.1 |
| 8. Lake area | 418.0 | 418.0 | 0.0 | 405.1 | 405.1 | 0.0 | 0.9 | 0.9 | 0.0 | 11.9 | 11.9 | 0.0 |
| 9. Bare land | 1327.1 | 1335.4 | 0.6 | 743.5 | 731.0 | -1.7 | 100.8 | 105.1 | 4.3 | 482.8 | 499.2 | 3.4 |
| 10.Cultivated land | 84.9 | 6.3 | -92.6 | 0.0 | 0.0 | - | 0.0 | 0.0 | - | 84.9 | 6.3 | -92.6 |
| 11.Orchard | 12.9 | 12.4 | -3.9 | 0.0 | 0.0 | - | 0.0 | 0.0 | - | 12.9 | 12.4 | -3.9 |
| 12.Commerce and service land | 2.6 | 13.4 | 415.4 | 0.0 | 0.0 | - | 0.0 | 0.0 | - | 2.6 | 13.4 | 415.4 |
| 13.Industry and storage land | 22.7 | 3.3 | -85.5 | 0.2 | 0.0 | -100.0 | 2.1 | 0.0 | -100.0 | 10.5 | 0.0 | -100 |
| 14.Residential land | 3.3 | 0.0 | -100.0 | 0.0 | 0.0 | - | 0.0 | 0.0 | - | 3.3 | 0.0 | -100.0 |
| 15.Scenic facilities | 1.7 | 15.4 | 805.9 | 0.0 | 0.3 | - | 0.0 | 2.1 | - | 1.7 | 13.0 | 664.7 |
| 16.Public facilities | 46.7 | 59.9 | 28.3 | 0.0 | 0.2 | - | 0.0 | 0.0 | - | 46.7 | 59.8 | 28.1 |
| 17.Special purposes land | 17.7 | 20.1 | 13.6 | 1.9 | 1.9 | 0.0 | 0.0 | 0.0 | - | 15.8 | 18.2 | 15.2 |
| 18.Highways | 89.0 | 103.1 | 15.8 | 23.7 | 3.5 | -85.2 | 32.0 | 24.7 | -22.9 | 33.3 | 73.8 | 121.7 |
| 19.Forest roads | 86.7 | 76.1 | -12.2 | 47.8 | 51.4 | 7.5 | 12.9 | 12.9 | 0.0 | 25.3 | 11.8 | -53.4 |
| 20.Ditches | 3.3 | 3.3 | 0.0 | 0.0 | 0.0 | - | 0.0 | 0.0 | - | 3.3 | 3.3 | 0.0 |
| 21.Hydraulic architectures | 8.5 | 6.8 | -20.0 | 0.5 | 0.5 | 0.0 | 0.0 | 0.0 | - | 8.0 | 6.3 | -21.2 |
| 22.Facility agricultural land | 0.0 | 0.6 | - | 0.0 | 0.0 | - | 0.0 | 0.0 | - | 0.0 | 0.6 | - |
