# Supplementary material for: The Efficacy of Landscape-Level Conservation in Changbai Mountain Biosphere Reserve, China
Source: PLoS One. 2014 Apr 17;9(4):e95081. doi: 10.1371/journal.pone.0095081 (PMC3990618; doi:10.1371/journal.pone.0095081)
Supplement: File S1 — Table S1 Classification accuracy of each type of natural landscape in 2012. Table S2 Spatial pattern characteristics of protected landscape mosaic. Table S3 Variation of area of landscape in CMBR and in each functional zone from 1993 to 2012. Table S4 Area transfer matrix of landscape type in CMBR from 1993 to 2012. Text S1 Specific processes of classification for natural landscape. Text S2 Equations of pattern indexes. (ZIP) [file pone.0095081.s001.zip › Table S4.doc]

**Table S4.** Area transfer matrix of landscape type in CMBR from 1993 to 2012	unit: ha

|  | 01 | 02 | 03 | 04 | 05 | 07 | 08 | 09 | 10 | 11 | 12 | 13 | 15 | 16 | 17 | 18 | 19 | 20 | 21 | 22 |
| --- | --- | --- | --- | --- | --- | --- | --- | --- | --- | --- | --- | --- | --- | --- | --- | --- | --- | --- | --- | --- |
| 01 | 167957.2 | - | 521.9 | 27.5 | - | 152.3 | - | 5.2 | - | - | 2.4 | - | 2.1 | 2.3 | 2.3 | 19.2 | 0.0 | - | 0.1 | - |
| 02 | - | 224.3 | - | - | - | - | - | - | - | - | - | - | - | - | - | - | 0.3 | - | - | - |
| 03 | 1555.1 | - | 5031.6 | 34.8 | - | 878.6 | - | - | - | - | - | - | 1.8 |  | - | 3.3 | - | - | - | - |
| 04 | 34.9 | - | 86.5 | 4743.0 | - | 16.4 | - | 17.4 | - | - | - | - | 0.6 | - | 1.1 | 2.5 | - | - | - | - |
| 05 | - | 1863.6 | - | - | - | - | - | - | - | - | - | - | - | - | - | 4.3 | 6.1 | - | - | - |
| 06 | 1300.9 | - | 1140.4 | 3.7 | - | 206.6 | - | - | - | - | - | - | - | - | - | 1.9 | - | - | - | - |
| 07 | 11.0 | - | 1181.1 | 14.3 | - | 7029.6 | - | 5.9 | - | - | - | - | - | - | - | 3.4 | - | - | - | - |
| 08 | - | - | - | - | - | - | 418.0 | - | - | - | - | - | - | - | - | - | - | - | - | - |
| 09 | 0.3 | - | - | 20.4 | - | 0.1 | - | - | - | - | 0.7 | - | - | 0.1 | - | 0.0 | - | - | - | - |
| 10 | 3.4 | 48.1 | 4.0 | 13.7 | 5.8 | 0.2 | - | - | 4.6 | - | - | - | - | 5.3 | - | - | - | - | - | - |
| 11 | 0.0 | - | - | - | - | - | - | - | - | 12.3 | - | - | - | - | - | - | - | - | - | 0.6 |
| 12 | - | - | - | - | - | - | - | - | - | - | 2.2 | - | 0.4 | - | - | 0.0 | - | - | - | - |
| 13 | - | - | - | - | - | - | - | - | 1.7 | - | 2.7 | 3.3 | 8.6 | 6.3 | - | 0.1 | - | - | - | - |
| 14 | - | - | - | - | - | - | - | - | - | - | 3.3 | - | - | - | - | - | - | - | - | - |
| 15 | - | - | - | - | - | - | - | - | - | - | - | - | 1.7 | - | - | 0.0 | - | - | - | - |
| 16 | - | - | - | - | - | - | - | 0.2 | - | - | 2.2 | - | - | 44.3 | - | 0.0 | - | - | - | - |
| 17 | 0.0 | - | - | - | - | - | - | 1.1 | - | - | - | - | - | - | 16.6 | - | - | - | - | - |
| 18 | 35.3 | - | 2.6 | 0.0 | - | - | - | 0.2 | - | - | 0.0 | - | 0.2 | - | - | 50.7 | 0.0 | - | - | - |
| 19 | - | - | - | - | - | - | - | - | - | - | - | - | - | - | - | 17.1 | 69.7 | - | - | - |
| 20 | - | - | - | - | - | - | - | - | - | - | - | - | - | - | - | - | - | 3.3 | - | - |
| 21 | 0.0 | - | - | - | - | 0.0 | - | - | - | - | - | - | - | 1.8 | - | - | - | - | 6.7 | - |

The serial numbers from 1 to 22 are the same as those in **Table S3**, represent the types of landscape.
