# Supplementary material for: The Efficacy of Landscape-Level Conservation in Changbai Mountain Biosphere Reserve, China
Source: PLoS One. 2014 Apr 17;9(4):e95081. doi: 10.1371/journal.pone.0095081 (PMC3990618; doi:10.1371/journal.pone.0095081)
Supplement: File S1 — Table S1 Classification accuracy of each type of natural landscape in 2012. Table S2 Spatial pattern characteristics of protected landscape mosaic. Table S3 Variation of area of landscape in CMBR and in each functional zone from 1993 to 2012. Table S4 Area transfer matrix of landscape type in CMBR from 1993 to 2012. Text S1 Specific processes of classification for natural landscape. Text S2 Equations of pattern indexes. (ZIP) [file pone.0095081.s001.zip › Text S1.doc]

**Text S1.** Specific processes of classification for natural landscape

First, the Edge-Based Segmentation algorithm was used, i.e. after repeated experiments the threshold of segmentation was set to 45, and the better edge characteristics could be obtained by segmentation. Second, the Full Lambda-Schedule algorithm was used, the threshold of mergence was set to 90 and then adjacent small patches were iteratively merged based on spectral and spatial information. Third, the attributes of image space, spectrum, texture, color space and band ratio (NDVI) were calculated. Fourth, the classification samples of the natural landscape (natural forest, sparse forest, shrub forest, grassland, lake area, and bare land) were selected based on the field data collected in August 2010. Fifth, the K Nearest Neighbor method was used in the classification algorithm and images classified considering the adjacent 5 pixels. Sixth, the clustering and filtering method was used to remove background noise. Any regional pixels wrongly classified or missed were manually corrected based on the field survey data. Seventh, the confusion matrix was computed using the validation samples selected from the ZY-1 02C fusion image and HR panchromatic image, and the classification accuracy was tested.
