# Supplementary material for: The Efficacy of Landscape-Level Conservation in Changbai Mountain Biosphere Reserve, China
Source: PLoS One. 2014 Apr 17;9(4):e95081. doi: 10.1371/journal.pone.0095081 (PMC3990618; doi:10.1371/journal.pone.0095081)
Supplement: File S1 — Table S1 Classification accuracy of each type of natural landscape in 2012. Table S2 Spatial pattern characteristics of protected landscape mosaic. Table S3 Variation of area of landscape in CMBR and in each functional zone from 1993 to 2012. Table S4 Area transfer matrix of landscape type in CMBR from 1993 to 2012. Text S1 Specific processes of classification for natural landscape. Text S2 Equations of pattern indexes. (ZIP) [file pone.0095081.s001.zip › Text S2.doc]

**Text S2.** Equations of pattern indexes

The pattern indexes include the number of patches (*NP*), edge density (*ED*), perimeter-area fractal dimension (*FR*), division index (*DI*), and connectivity index (*CON*) as follows:

$$NP=\text{n}_{\text{i}}$$

where *n_i_* is the number of patches of class *i* landscape.

$$ED=\frac{\sum_{\text{j}=1}^{\text{n}} \text{e}_{\text{ij}}}{a_{i}}$$

where $e_{ij}$ is the total length (m) of edge in landscape involving patch type (class) *i*; includes landscape boundary and background segments involving patch type *I*; $a_{i}$ is the total area (ha) of the type *i*.

$$FR=\frac{2}{\frac{n\sum_{j=1}^{n} \text{lnp}_{\text{ij}}\text{lna}_{\text{ij}}\text{-}\sum_{j=1}^{n} \text{lnp}_{\text{ij}}\sum_{j=1}^{n} \ln a_{ij}}{n\sum_{j=1}^{n} \text{lnp}_{\text{ij}}^{2}-(\sum_{j=1}^{n} \text{lnp}_{\text{ij}})^{2}}}$$

where $p_{ij}$ is the Perimeter (m) of the patch *ij*; $\text{a}_{ij}$ is the area of the patch *ij*; *FR* equals 2 divided by the slope of regression line obtained by regressing the logarithm of patch area (m^2^) against the logarithm of patch perimeter (m). $1\leq FR\leq2$, *FR* approaches 1 for shapes with very simple perimeters such as squares, and approaches 2 for shapes with highly convoluted, plane-filling perimeters.

$$DI=1\text{-}\sum_{\text{j}=1}^{\text{n}} (\frac{a_{ij}}{a_{i}})^{2}$$

where $\text{a}_{ij}$ is the area of the patch *ij*，$a_{i}$ is the total area of the patch type *i*. $0\leq DI<1$, *DI* = 0 when the landscape consists of single patch. *DI* approaches 1 when the focal patch type consists of single, small patch one cell in area. As the proportion of the landscape comprised of the focal patch type decreases, and as those patches decrease in size, *DI* approaches 1.

$$CON=\frac{\sum_{\text{j}\neq\text{k}}^{\text{n}} \text{c}_{\text{ijk}}}{\frac{\text{n}_{\text{i}}\text{(n}_{\text{i}}\text{-}1)}{2}}$$

where *c_ijk_* is the number of the joining between patch *j* and *k* of the corresponding patch type *i*, based on a specified threshold distance. *n_i_* is the number of patches in the landscape of the corresponding patch type. $0\leq CON\leq100$, *CON* = 0 when either the focal class consists of a single patch or none of the patches of the focal class are considered to be connected (i.e., within the specified threshold distance of another patch of the same type). *CON* = 100 when every patch of the focal class is considered to be connected.
